# Prosthetic valve detachment complicated with intervalvular fibrous body destruction in Behcet’s disease: a case report

**DOI:** 10.1186/s12893-021-01164-9

**Published:** 2021-03-25

**Authors:** Xiaoli Qin, Weitao Liang, Honghua Yue, Zhong Wu

**Affiliations:** Department of Cardiovascular Surgery, West China Hospital, Sichuan University, Sichuan 610000 Chengdu, People’s Republic of China

**Keywords:** Behcet’s disease, Intervalvular fibrous body, Valve detachment

## Abstract

**Background:**

Prosthetic valve detachment is not rare after aortic valve replacement in Behcet’s disease. However, destruction of the intervalvular fibrous body (IFB) due to Behcet’s disease was rarely reported.

**Case presentation:**

We report a case of 30 year-old woman, with valve detachment and IFB separation. The patient received aortic valve replacement three months ago. Her medical history included recurrent oral ulcers and cutaneous lesions. Finally, reoperation was performed and peri-operative steroid therapy was carried out.

**Conclusions:**

The case presented a rare complication of valve detachment in Behcet’s disease.

## Background

Aortic regurgitation caused by Behcet’s disease often need surgical treatment [1]. There is a high risk of prosthetic valve detachment and perivalvular leakage after aortic valve replacement (AVR) in Behcet’s disease [2]. However, destruction of the intervalvular fibrous body (IFB) is rare. We report a case of valve detachment complicated with IFB separation due to Behcet’s disease.

## Case presentation

**Fig. 1:**
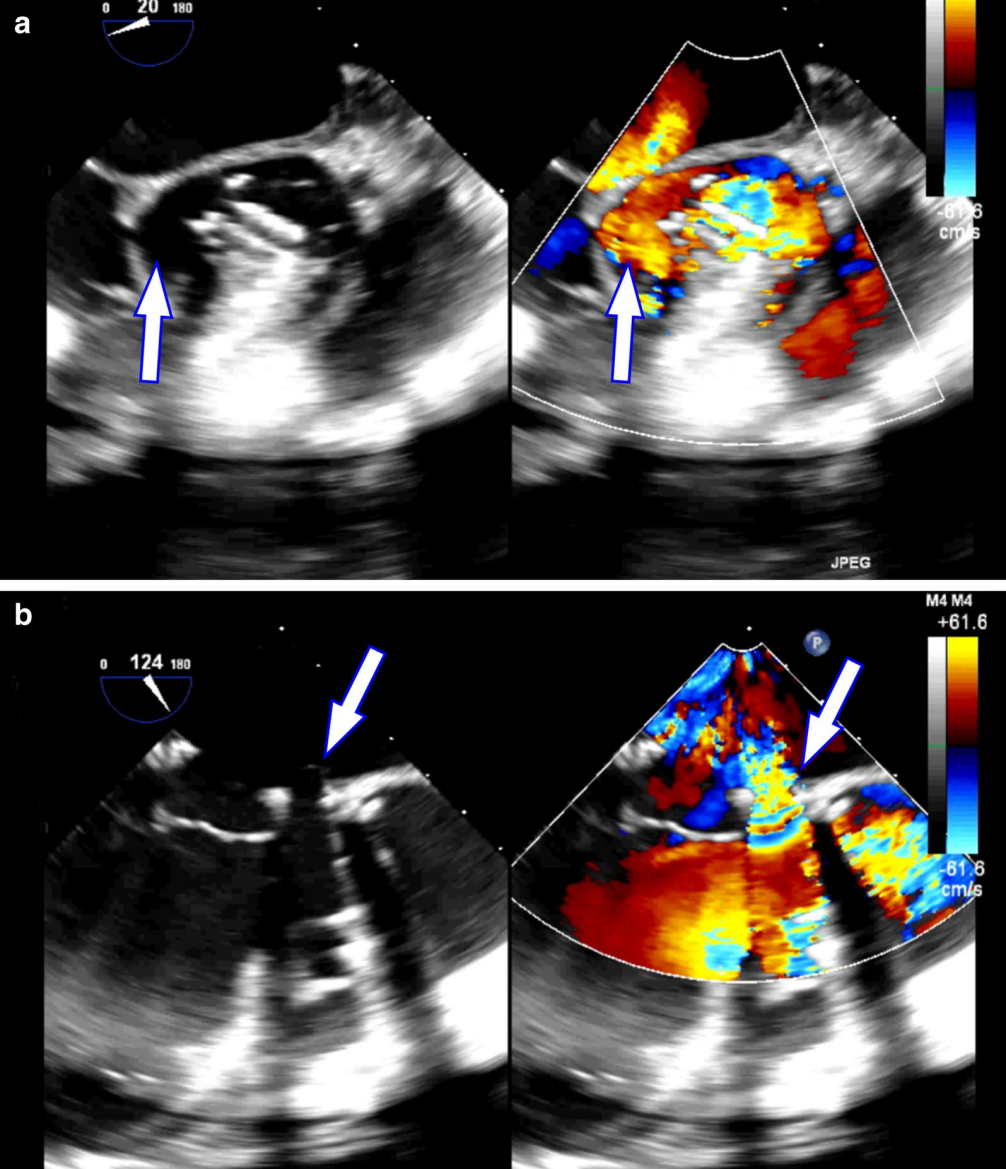
Echocardiography revealed prosthetic valve detachment, severe perivalvular leakage (**a**) and aorto–mitral curtain separation (**b**)

A 30-year-old woman was transferred to the emergency department from another hospital. The patient underwent aortic valve replacement and mitral valvuloplasty to treat moderate aortic regurgitation and mitral regurgitation three months ago. She presented with chest tightness and dyspnea for one week and echocardiography revealed prosthetic valve detachment, severe perivalvular leakage and moderate mitral regurgitation with posterior eccentric jet (Fig. 1). Her medical history included recurrent oral ulcers and cutaneous lesions. Based on these findings, a diagnosis of Behcet’s disease was considered. Erythrocyte sedimentation rate (ESR) was 74 mm/h and C-reactive protein (CRP) was 29.3 mg/L. The patient’s condition was suspected as being in the active phase, so methylprednisolone therapy (40 mg/day) and thalidomide (immunomodulators, 50 mg/day) were initiated. Through 10 days drug therapy, ESR decreased to 39 mm/h and CRP to 6.04 mg/L.

**Fig. 2:**
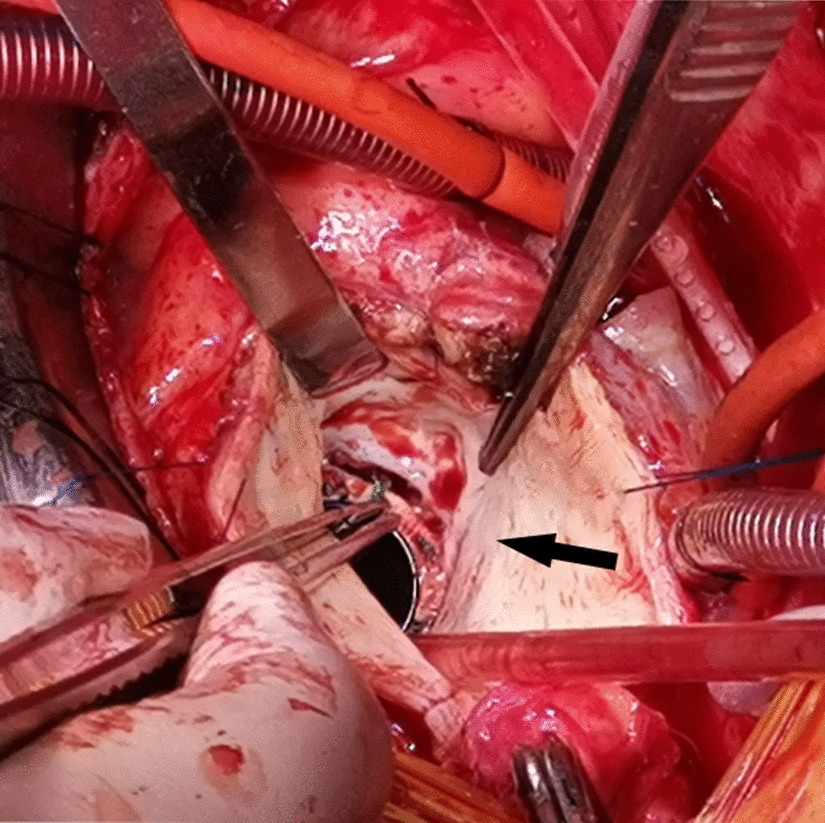
Intraoperative image showing the prosthetic valve detachment near the noncoronary sinus of Valsalva

**Fig. 3:**
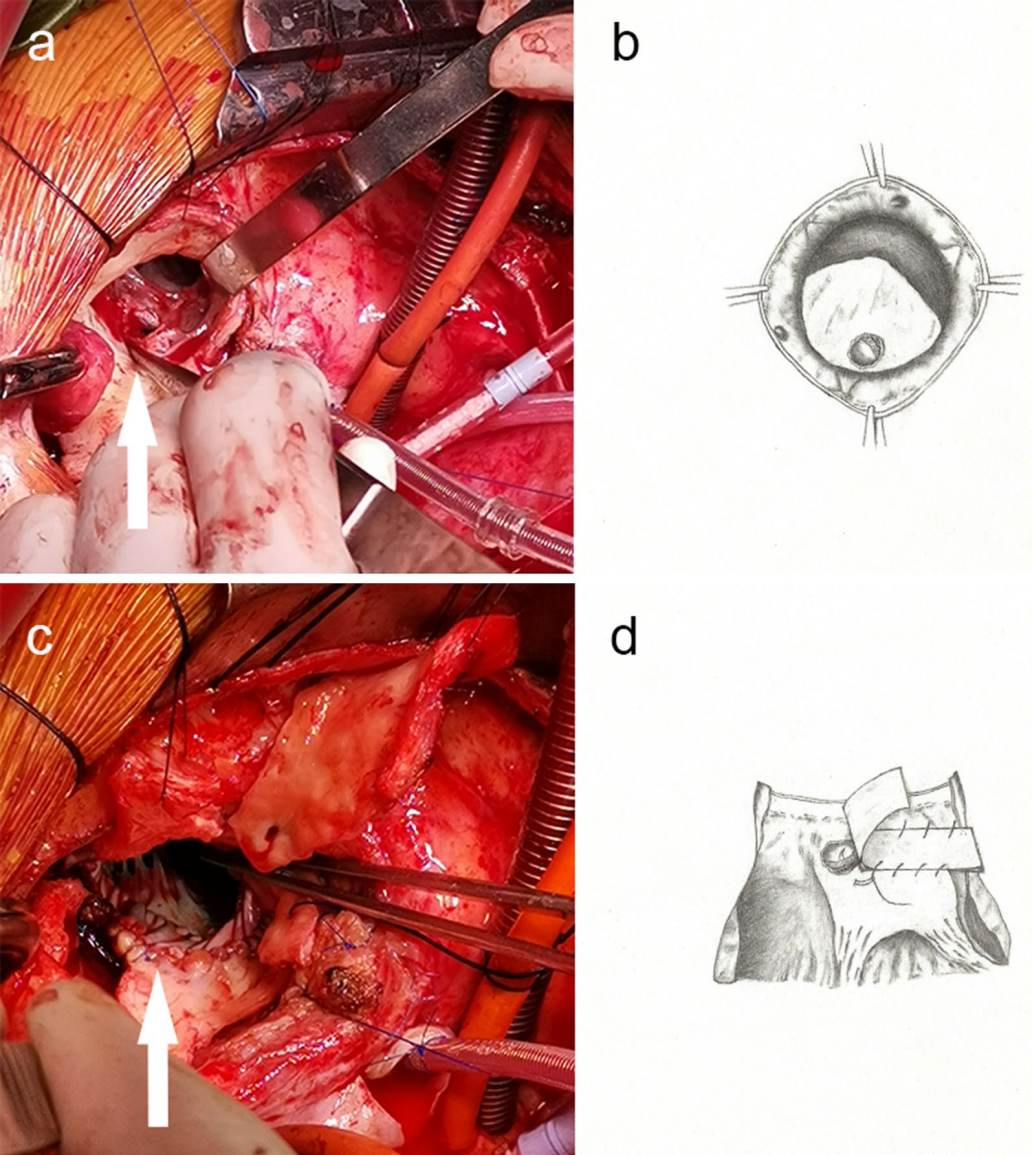
The prosthetic valve was removed and there was a 1 cm-long tear in the mitral-aortic intervalvular fibrous body (**a**, **b**). A bovine pericardial patch was sutured to aorto-mitral curtain and the posterior segment of the left ventricular outflow tract (**c**, **d**)

During surgery, a median sternotomy was performed. Cardiopulmonary bypass was established with femoral arterial and central bicaval cannulation. Myocardial protection was achieved using cold antegrade and retrograde crystalloid cardioplegia. After opening the ascending aorta, the aortic wall was thickened, and the valve detachment accounted for approximately half of the annulus (Fig. 2). The prosthetic valve was removed and there was a 1 cm-long tear in the aorto-mitral IFB (Fig. 3a, b). The aortic root and surrounding tissues were fragile. The thickened aortic root was resected and the coronary buttons were constructed with a 0.5 to 0.8 cm diameter cuff of aortic wall and mobilized over a short length to facilitate reimplantation. We reconstructed the left ventricular outflow tract and the aortic annulus using a tailored single bovine pericardial patch and repaired the mitral-aortic IFB (Fig. 3c, d). The valved conduit was implanted in the aortic annulus with a belt-like Teflon felt interrupted mattress sutured outside. Then, the coronary buttons were reimplanted to the valved conduit without tension. Finally, the distal end of the valved conduit was anastomosed to the distal ascending aorta. Postoperative glucocorticoid and thalidomide therapy were continued. The patient had an uncomplicated postoperative course and was discharged on the 10th day after the surgery. Histologic examinations of the aortic walls indicated disruption of medial elastic membrane, myxoid degeneration and lymphocyte infiltration.

## Discussion and conclusions

Behcet’s disease is a systemic disorder characterized by recurrent aphthous stomatitis, genital ulceration, and iridocyclitis [1]. A new diagnosis criterion was recently proposed by a collaborative study of 27 countries [3]. Cardiovascular involvement in Behçet’s disease is serious and its incidence is little documented.

Isolated AVR in Behcet’s disease is often complicated with valve detachment and perivalvular leakage. Both the aortic annulus and the ascending aortic wall are fragile because of recurrent inflammation, so postoperative complications such as recurrent valve detachment and pseudoaneurysm are not rare [2]. However, destruction of IFB after AVR in Behcet’s disease was rarely reported in the literature.

It may be misdiagnosed as infective endocarditis [4], especially when patient presented with fever and chill [5]. In the present case, we suspected infective endocarditis but the patient had never had a fever and repeated blood cultures were negative several times. In addition, no vegetation was found during the operation. The perivalvar tissue culture was performed and the result was negative.

Perioperative glucocorticoid administration therapy may be beneficial [2, 6]. However, there is no standard for the dosage and duration. Preoperative glucocorticoid therapy is used to suppress the active inflammatory reaction. ESR and CRP are the makers to reflect inflammatory activity, which is helpful for determining the timing of surgery and evaluating the effect of drug therapy. Postoperative glucocorticoid therapy is necessary. In a long-term observation study, postoperative inflammatory markers were negatively correlated with freedom from cardiac reoperation and death during follow-up [2]. Recently, another study reported that lower CRP levels maintained through glucocorticoid and immunomodulation therapy were associated with better surgical outcomes [6]. Previous studies also revealed improved outcomes was associated with the use of immunomodulators [7].

Several surgical modifications have been described to prevent the devastating postoperative complications, including the use of aortic root replacement and modified Bentall procedure [8, 9]. As for valve detachment after AVR, surgical intervention may be more complicated. Yangfeng Tang and colleagues have recommended a supraannular aortic valve replacement to rescue the valve detachment after AVR attributable to Behçet’s disease [10]. They used interrupted mattress sutures buttressed to a Teflon felt strip on the lateral side of the aortic wall for reinforcement and placed the prosthetic valve between the native annulus and the coronary ostium line.

According to the study by Liangwan Chen et al. [11], the left ventricular outflow tract myocardial tissue was free of inflammatory involvement. They directly sutured a valved conduit to the left ventricular outflow tract other than the aortic annulus. In our case, the native aortic annulus and IFB were destructed. We reconstructed the fragile aortic annulus and repaired the IFB with a pericardial patch. Moreover, we placed Teflon felt strip on the lateral side of conduit. The “sandwich” technique may be beneficial in preventing pseudoaneurysm.

This case reported that IFB destruction is a rare complication of prosthetic valve detachment in Behcet’s disease. It is easy to be misdiagnosed when the characteristics of Behcet’s disease are not typical. Glucocorticoid therapy and surgical modification are of great significance in preventing recurrent valve detachment and pseudoaneurysm.

## Data Availability

Not applicable.

## Abbreviations

AVR: Aortic valve replacement
IFB: Intervalvular fibrous body
ESR: Erythrocyte sedimentation rate
CRP: C-reactive protein

## References

[1] Erentug V, Polat A, Bozbuga NU, Erdogan HB, Ozkaynak B, Akinci E (2006). Valvular surgery in Behcet’s disease. J Card Surg.

[2] Jeong DS, Kim KH, Kim JS, Ahn H (2009). Long-term experience of surgical treatment for aortic regurgitation attributable to Behçet’s disease. Ann Thorac Surg.

[3] Davatchi F, Assaad-Khalil S, Calamia KT, Crook JE, Sadeghi-Abdollahi B, Schirmer M (2014). The International Criteria for Behçet’s Disease (ICBD): a collaborative study of 27 countries on the sensitivity and specificity of the new criteria. J Eur Acad Dermatol Venereol.

[4] Han JK, Kim HK, Kim YJ, Cho GY, Kim MA, Sohn DW (2009). Behçet’s disease as a frequently unrecognized cause of aortic regurgitation: suggestive and misleading echocardiography findings. J Am Soc Echocardiogr.

[5] Zou Y, Ni Y, Liu X, Chen X (2012). Misdiagnosis of Behçet’s disease with unknown protracted fever and chill after surgical excision of cardiac tumor. Rheumatol Int.

[6] Ghang B, Kim JB, Jung SH, Chung CH, Lee JW, Song JM (2019). Surgical outcomes in Behcet’s disease patients with severe aortic regurgitation. Ann Thorac Surg.

[7] Hatemi G, Silman A, Bang D, Bodaghi B, Chamberlain AM, Gul A (2008). EULAR recommendations for the management of Behçet disease. Ann Rheum Dis.

[8] Yoshikawa K, Hori H, Fukunaga S, Tayama E, Aoyagi S (2007). Aortic root replacement in Behçet disease. Asian Cardiovasc Thorac Ann.

[9] Azuma T, Yamazaki K, Saito S, Kurosawa H (2009). Aortic valve replacement in Behcet’s disease: surgical modification to prevent valve detachment. Eur J Cardiothorac Surg.

[10] Tang Y, Xu Z, Liao Z, Xu J (2012). Supraannular aortic replacement for severe valve detachment attributable to Behçet’s disease. Ann Thorac Surg.

[11] Chen LW, Wu XJ, Cao H, Dai XF (2017). Valved conduit attached to left ventricular outflow tract for valve detachment in Behçet’s disease. Ann Thorac Surg.

